# Effects of Sanguinary Deposits on the Teeth

**Published:** 1891-05

**Authors:** B. F. Arrington

**Affiliations:** Asheville, N. C.


ARTICLE II.

### EFFECTS OF SANGUINARY DEPOSITS ON THE TEETH.

BY B. F. ARRINGTON, D. D. S., of ASHEVILLE, N. C.

Having furnished the AMERICAN JOURNAL OF DENTAL SCIENCE a paper on pyorrhea alveolaris and in that paper drew the distinction between pyorrhea trouble and that caused by serumal or sanguinary deposits, I herewith inclose to you, and for your careful inspection, two specimens of sanguinary deposits on teeth, which I was necessitated to extract for relief and cure after several months' treatment, and repeated efforts (ineffectual) to dislodge and remove the deposits. Having failed in my efforts for relief and cure, I advised extraction and realized immediately upon examination that I had diagnosed and advised correctly, as you will, I think, decide, when you have carefully examined with a good magnifying glass, the deposits on the roots of the teeth, and read my story as regards No. 1, the superior molar. The patient, male, about 40 years of age, came to me about six months ago to consult in relation to the tooth, saying it felt very uncomfortable at times and he conceived the idea that it was a little loose at times. I made a careful inspection of the tooth and teeth adjoining, and could find no defect worthy of attention and treatment. The crown of the tooth, as you can see, was in a comparatively normal condition, with the exception of a slight decay confined to the enamel and the calcarious deposit, the latter not at all perceptible, the entire crown to the margin of the gum was clear and free from deposit, and for a month or two afterwards he ceased chewing and masticating food on the right side, as he informed me, in consequence of soreness and discomfort of tooth under pressure. The calcarious deposit on crown very unlike the deposits on roots, and evidently the effect of neglected use of the tooth in masticating. The gum around the neck of the tooth at first examination was normal; never examined a gum that was seemingly more healthy, and so continued for more than two months, and the entire gum covering roots and surrounding adjoining teeth seemed to be in a perfectly normal condition. During the first ten weeks of treatment, I did nothing more than apply occasionally, with pencil brush, to the gum surrounding the tooth iodine and tincture of aconite, equal parts, hoping through such means to be able to subdue what seemed to be a simple case of inflammation about the roots of the tooth. Two weeks later patient returned complaining of increased discomfort. Upon inspection I found the margin of the gum in a condition to justify the impression that pus had formed; placing my finger near the point of palatal root and pressing forcibly towards crown of tooth caused a free discharge of pus at margin of gum. I then introduced instruments for removal of detected deposits, and repeated the effort with that object in view a half dozen times or more, thinking each time I had probably removed every particle of deposit, but would learn very soon from other evidences of continued trouble that I had not. I tried faithfully the application of sulphuric acid, one of acid to five, seven or ten of water, as best I could apply with hypodermic syringe, and floating it around the neck of tooth and down into the pus pocket with pencil brush, but to no effect. The trouble grew worse and worse until three days ago I advised extraction, and found the tooth, as you see it, and to see it rightly and be able to appreciate the extent of deposit you must use a strong magnifying glass with good light. Points of roots denuded slightly, but to the extent of nerve destruction as is often the case. When I split the tooth open, in presence of patient, it was most offensive, the pulp a mass of pus. No. 2 was extracted on first visit of patient and in less time than two minutes from first inspection of tooth. It was a plain case and would not admit of treatment, because patient lived considerable distance in the country. Extraction in such cases is always wisest, and gives greatest satisfaction to patients. By some dentists both of these cases would be termed pyorrhea alveolaris, and treated accordingly. No symptom of pyorrhea in either case.

